# Authors’ Response to Peer Reviews of “A Local Community-Based Social Network for Mental Health and Well-being (Quokka): Exploratory Feasibility Study”

**DOI:** 10.2196/33199

**Published:** 2021-10-27

**Authors:** Cynthia Shih, Ruhi Pudipeddi, Arany Uthayakumar, Peter Washington

**Affiliations:** 1 Quokka Palo Alto, CA United States; 2 Department of Computer Science University of California, Berkeley Berkeley, CA United States; 3 Department of Cognitive Science University of California, Berkeley Berkeley, CA United States; 4 Department of Bioengineering Stanford University Stanford, CA United States

**Keywords:** local social network, community health, well-being, digital health, consumer health

*This is the authors’ response to peer-review reports for “A Local Community-Based Social Network for Mental Health and Well-being (Quokka): Exploratory Feasibility Study.”*

## Round 1 Review

We would like to thank the Editor and Reviewers for excellent comments that have vastly improved the quality of our manuscript [1]. Below, we provide a point-by-point response to reviewers, and we address all points made by all reviewers.

### Anonymous Reviewer [2]

1. We thank the anonymous reviewer for pointing out this omission. We have added an entire subsection to the *Methods* section, titled *Quokka System*, which explicitly describes the components of the Quokka system in more detail.

2. We apologize for this confusion, and we should have been more careful when originally reporting our results. We meant to convey that we reject the NULL hypothesis that similar proportions of users would participate in local and remote activities during the challenges. Our alternate hypothesis, that users prefer local over remote activities, was therefore confirmed. We have clarified this language throughout the manuscript and have explicitly referred to the hypotheses as H1, H2, and H3.

3. We thank the anonymous reviewer for finding this lack of clarity. We have added a new section to the *Methods*, called *Statistical Tests*, which provides details about how we tested for significance. In particular, we added: “To perform statistical testing for H1-H3, we conducted a binomial proportion test, where we used the proportion of local (H1), social (H2), and new (H3) self-reported activities per week. The null hypothesis is that the proportion is 0.5 (equal number of local and remote, social and individual, as well as new and familiar activities). The goal was to determine if the increased rates of local, social, and new self-reported activities was statistically significant. We calculated a Clopper-Pearson Binomial proportion confidence interval for one test, a method which leverages the cumulative probabilities of the binomial distribution.”

4. We have clarified these points throughout the manuscript. We chose the local versus social, remote versus in-person, and familiar versus new categories because these were the unique aspects of Quokka that we wanted to test. Other health and wellness digital health tools do not focus on the social, in-person, and novel experience aspects of well-being, and we wanted to ensure that Quokka would feasibly promote increased activity of these kinds. In order to code these categories from participant questionnaire responses, we hired 3 independent raters recruited on Upwork, a popular web-based freelancing platform that connects workers to job providers. To reach the final category, a majority-rules consensus was taken for the categorical labels provided by raters. In cases where all 3 raters disagreed, the authors provided the final rating. Protected user data were anonymized when provided to Upwork workers. We have updated the manuscript with these details.

5. We have added a new section to our manuscript, called *Limitations: Study Design*, which highlights the limitation of the analysis consisting of quantitative analysis of coded qualitative data.

6. Because this research was conducted outside of our capacity as students at a University, and our startup company does not have an ethics review committee, no formal University ethics review was sought for this study. However, all users provided informed consent for participation. In particular, all participants consented on signup to a Privacy Policy and a Terms and Conditions that walked through how their data would be used. We ensured that all user data were anonymized during our analyses, and we do not reveal any protected health information or any other identifiable information in the manuscript. We believe that we fulfill JMIR’s requirements for ethics approval [3]. We have added to the manuscript that the research was conducted in accordance with the Declaration of Helsinki.

We are happy to work with the Editor to ensure that this manuscript can fulfill the ethics review requirements on JMIR. Please let us know if there are any additional steps we should take to address this concern.

### Reviewer J

1. We thank Reviewer J [4] for pointing out this lack of clarity. The challenges were part of the interventional program that makes use of the established success of community-based social programs for behavior change, in particular through a new community-based social network, Quokka, which was the embodiment of the interventional paradigm we were exploring the feasibility of. We have updated the manuscript to reflect this more clearly.

2. The program focused on one habit per week, although participants were encouraged to stick to whichever habits they found most effective both through the duration of the program and afterwards. A final survey was sent to participants at the end of each program to collect input and feedback from participants. Respondents cited which habits they had continued and were planning to continue from then on, although this was not further assessed after the program completion.

3. We reached out to over 15 US colleges and universities and met with several administrative health services and student health club staff in order to discuss the possibility of running a program on their campuses. Since this was an early pilot, we chose a small subset of schools to coordinate programs based on their overall interest and availability in dedicating time and effort towards participating. We have clarified this point in the manuscript.

4. We believe that the confirmation of the feasibility of Quokka to promote local, social, and novel experiences can be generalized to other universities, particularly given the differences in student body size and demographics between the schools.

Typical demographics of college students in the United States follow an approximately equal split of women and men (with women holding a slight majority). The vast majority of these students are between 18 and 24 years old (87.5% in 2017, when this study took place). By ethnicity, the undergraduate college student population in 2017 was approximately 53% non-Hispanic White, 21% Hispanic, 15% Black, 8% Asian, and 3% non-Hispanic “Other” [5].

For the four universities included in this study, the typical demographics of their undergraduate college students followed a similar pattern to the national statistics: approximately equal split between women/men, primarily between 18 and 24 years old, and predominantly identifying as non-Hispanic White with varying distributions of students identifying as Hispanic, Black, Asian, or “Other.”

We have included this information in the manuscript.

5. We reached out to administrative health services and student health clubs on each campus, meeting with prospective candidates who would be interested in volunteering. Typically, one student health club or group per campus would become the designated “host” while working with other school resources and groups to customize their programs. We have added this information to the manuscript.

6. Typical demographics of college students in the United States follow an approximately equal split of women and men (with women holding a slight majority). The vast majority of these students are between 18 and 24 years old (87.5% in 2017, when this study took place). By ethnicity, the undergraduate college student population in 2017 was approximately 53% non-Hispanic White, 21% Hispanic, 15% Black, 8% Asian, and 3% non-Hispanic “Other” [5].

For the four universities included in this study, the typical demographics of their undergraduate college students followed a similar pattern to the national statistics: approximately equal split between women/men, primarily between 18 and 24 years old, and predominantly identifying as non-Hispanic White with varying distributions of students identifying as Hispanic, Black, Asian, or “Other.”

We have included this information in the manuscript.

7. We thank Reviewer J for identifying these areas for improvement. We have clarified all of these issues throughout the manuscript, and we refer to explicit hypotheses throughout.

8. We have reviewed the manuscript with an eye towards substituting imprecise terms throughout the manuscript.

9. We have now added section headers for the challenges list in the *Challenges Themes* section.

### Reviewer K

1. We agree with this point [6], and we concur that an ideal study would have included outcome measures relating to well-being. Because such an analysis was missing, we have limited the scope of this manuscript to be an “exploratory feasibility study” with the aim of evaluating the Quokka system’s potential for promotion of local, social, and unfamiliar activities as they pertain to healthy habits. A larger, more controlled study with outcome measure tracking will be required to claim anything beyond an exploratory feasibility study.

2. The Quokka Challenge was designed as a new program in the fall academic quarter/semester of 2017 to promote healthier habits in the university setting. The program design and implementation were influenced by prior research in the field, although it was uniquely created for the university setting. This manuscript highlights Quokka’s first pilot programs, evaluating its framework’s potential for increasing participation in healthy habits.

3. The Quokka platform was designed by a team of engineers, including the authors of this manuscript, who designed every aspect of the platform and challenge. Additional names are listed in the manuscript’s *Acknowledgments* section.

4. We do not have an explanation for the low retention, other than that our program had higher retention than the average well-being digital application [7]. We have included in the manuscript a citation to this study about the user retention of digital wellbeing apps.

5. We did not employ a control group for this study. We agree that this is a limitation which prevents us from describing the presented study as anything beyond an “exploratory feasibility study.” We have emphasized throughout the manuscript that the presented study is only exploratory in nature, and we have emphasized in the *Limitations* that we do not know whether Quokka increases social, local, or new activities. We only know that when participating in the Quokka challenge, users are more likely to conduct social, local, or new activities than individual, remote, or familiar activities, by a large and statistically significant margin.

6. We thank Reviewer K for pointing out this omission, which we have corrected in the manuscript.

7. Because this research was conducted outside of our capacity as students at a University, and our startup company does not have an ethics review committee, no formal University ethics review was sought for this study. However, all users provided informed consent for participation. In particular, all participants consented on signup to a Privacy Policy and a Terms and Conditions which walked through how their data would be used. We ensured that all user data were anonymized during our analyses, and we do not reveal any protected health information or any other identifiable information in the manuscript. We believe that we fulfill JMIR’s requirements for ethics approval as outlined here [3]. We have added to the manuscript that the research was conducted in accordance with the Declaration of Helsinki.

We are happy to work with the Editor to ensure that this manuscript can fulfill JMIR’s ethics review requirements. Please let us know if there are any additional steps we should take to address this concern.

8. The program was conducted during the fall academic quarter/semester of 2017, and this detail has been added to the manuscript introduction.

### Reviewer L

1. We thank Reviewer L [8] for bringing up this point. The Quokka Challenge was designed as a new program in the fall academic quarter/semester of 2017 to promote healthier habits in the university setting. The program design and implementation were influenced by prior research in the field, although it was uniquely created for the university setting. This manuscript now highlights Quokka’s first pilot programs, evaluating its framework’s potential for increasing participation in healthy habits. We have added this information to the manuscript.

2. We thank Reviewer L for pointing out this omission in the manuscript. We have created an explicit *Related Work* subsection in the *Introduction* which discusses the present study in the context of prior works.

## Round 2 Review

### Anonymous Reviewer [2]

1. We appreciate this response from the anonymous reviewer, and we have extensively proofread our manuscript to minimize any writing errors. We have replaced all instances of “patient” with “user.”

### Reviewer J

1. We thank Reviewer J for this suggestion, and we have removed the *Related Work* section and integrated this material into the *Introduction* section.

2. We thank Reviewer J for pointing us to the JMIR Instructions for Authors, which we have read and more carefully adhered to in the currently submitted revision, including adding a *Comparison With Prior Work* subsection in the *Discussion* section.

3. We have identified prior studies discussed here and in the *Introduction*; we searched for “digital mental health intervention local community”, “digital mental health intervention online community”, “mental health social network”, and “digital mental health intervention local social network” on Google Scholar as well as the Journal of Medical Internet Research website. We have added this information to the manuscript.

4. We have selected the work currently in the *Related Work* section in the same way we identified works in the *Comparison With Prior Work* section, as described above. We have clarified this methodology in the manuscript.

5. We have updated the title to be “Exploratory Feasibility Study of Quokka: A Local Community-Based Social Network for Mental Health and Wellbeing.” This title now reflects the major mental health component of this work.

### Reviewer K

1. We greatly thank Reviewer K for the kind review.

### Reviewer M

1. We sincerely apologize for our omission of Reviewer M’s comments [9]. This was completely unintentional on our end and was due to a copying error. We are embarrassed by this mistake and we have ensured that all reviewer comments are properly addressed for this revision round. We thank Reviewer M and the Editor for allowing us to address these issues here.

2. We have checked similar papers published in JMIR and we believe that the updated manuscript adheres to the journal style.

3. We thank Reviewer M for pointing out these missing details in the *Introduction*. We have added a new introductory paragraph that motivates the problem that Quokka attempts to address, namely, the large mental health burden globally. We also discuss the current solutions to mental health care in the second paragraph, including the limitations of these approaches and why a guided yet remote digital health intervention could address these issues.

4. We agree, and we have moved this section down to the *Discussion* section.

5. We agree that this formatting was not ideal. To address this concern, we have incorporated the old *Related Work* section into the *Introduction* section, which now provides several references in the *Introduction* providing motivation for the work. In addition, we have significantly bolstered the citations when describing the motivation for Quokka and the current solutions which exist today.

6. We have moved these paragraphs to other sections of the paper and we have restructured the Introduction as requested by Reviewer M.

7. We thank Reviewer M for informing us about behavior changing theory (BCT) literature. We have added a section about BCT and prior related studies in the Introduction section (the fourth paragraph in the revised manuscript).

8. We thank Reviewer M for pointing out this omission. We have added a list of manuscripts and clarified that the prior work we are comparing against, and in fact all prior work in this space does not leverage both local health opportunities and community-based programming to drive behavior change in a single social network.

9. We have added an explicit description of the aim of this study in the second to last paragraph of the *Introduction* section.

10. We sincerely apologize for our omission of Reviewer M’s comments. This was completely unintentional on our end and was due to a copying error. We are embarrassed by this mistake and we have ensured that all reviewer comments are properly addressed for this revision round. We thank Reviewer M and the Editor for allowing us to address these issues here.

11. We have separated what was previously the *Methods* section into two sections. The first section, called *The Quokka Platform and Challenge*, describes the Quokka system and program, and the *Methods* section now starts with the *Recruitment* subsection.

12. We thank Reviewer M for pointing out our lack of clarity regarding the exact study design and the purpose of the study. We note that the primary goal of this study was not to provide a controlled trial or to claim that Quokka is an intervention. Our goal was to test the feasibility of such a system by verifying that study participants engage in the behaviors suggested by Quokka for the duration of the program. We have clarified in the manuscript all questions asked by Reviewer M. In particular:

We designed the study as a feasibility study with three central hypotheses: (1) users will spend more time on local over remote activities, (2) users will spend more time on novel over familiar experiences, and (3) users will spend more time on social over individual challenges.

We acquired users through several recruitment strategies (eg, emails, posters) at four separate universities.

We did not include any exclusion criteria besides being a student at one of the four target universities.

All users signed a Terms of Service and Privacy Policy providing study consent. All user data is fully anonymized.

Data were collected by a weekly check-in which consisted of a weekly email reminder to fill in a survey form embedded on the Quokka website. All responses were free-form, and we categorized the responses after the study. Details are provided in the Methods section.

The study took place during the fall quarter/semester of 2017 at the respective universities.

13. We appreciate this response, and we have added a paragraph (fourth paragraph in the *Introduction*) discussing the theories of behavior change that Quokka is based on. Theories of behavior change suggest that intervention effectiveness may be increased through the incorporation of social and cultural factors that also influence behavior [10,11,12]. These theories targeting the lifestyle focus on learning and decision-making to drive action and reflection, but understanding other factors such as individual beliefs, motivations, and the environment, are important for continued maintenance of health as well [13,14]. Examples of behavior change theories that examine these additional factors as applied to health outcomes include the health belief model (ie, behavior change is posited on barriers, benefits, self-efficacy, and threat) and the theory of planned behavior (ie, actions are driven by behavioral intent, subjective norms, and perceived behavioral control) [14,15,16]. Several of these have been in the university setting, which is especially pertinent given the Quokka setting. Quokka builds upon prior works by incorporating social, cultural, and local environmental elements into its framework and examining the effects of these community factors on individual action and reflection. Furthermore, Quokka utilizes several digital intervention techniques (including option-based, attribute-based, and goal-based techniques) that build upon these theories to drive further habit formation and maintenance [11].

14. We have ensured that the paper has a clear and distinct *Results* section. This *Results* section include the following subsections: *User Statistics* (quantitative), *Evaluation Outcomes* (quantitative), *Participation Due to Localized Social Influence* (qualitative), *Shared Experiences* (qualitative), *Local Community-Supported Resources* (qualitative), and *User Reflection* (qualitative).

15. We thank Reviewer M for pointing out this omission. We have added an explicit section comparing against prior work and have clarified that the prior work we are comparing against, and in fact all prior work in this space, does not leverage both local health opportunities and community-based programming to drive behavior change in a single social network.

16. We have ensured that the *Limitations* sections are succinct and discuss the points brought up by Reviewer M.

17. We thank Reviewer M for finding this error in the reference format. We have updated the references according to the provided suggestions and have ensured that our reference format matches that of papers previously published in JMIR.

18. We thank Reviewer M for finding this error in the reference format. We have updated the references according to the provided suggestions.

19. We thank Reviewer M for pointing this out. We have included “et al” for all papers with more than 6 people.

## Abbreviations

BCT: behavior changing theory
